# Sex-Dependent Effects of Cadmium Exposure in Early Life on Gut Microbiota and Fat Accumulation in Mice

**DOI:** 10.1289/EHP360

**Published:** 2016-09-16

**Authors:** Qian Ba, Mian Li, Peizhan Chen, Chao Huang, Xiaohua Duan, Lijun Lu, Jingquan Li, Ruiai Chu, Dong Xie, Haiyun Song, Yongning Wu, Hao Ying, Xudong Jia, Hui Wang

**Affiliations:** 1 Key Laboratory of Food Safety Research, Institute for Nutritional Sciences, Shanghai Institutes for Biological Sciences, Chinese Academy of Sciences, Shanghai, China; 2 Key Laboratory of Food Safety Risk Assessment, Ministry of Health, Beijing, China; 3 School of Life Science and Technology, ShanghaiTech University, Shanghai, China; 4 Xuhui Central Hospital, Shanghai Clinical Center, Chinese Academy of Sciences, Shanghai, China

## Abstract

**Background:**

Environmental cadmium, with a high average dietary intake, is a severe public health risk. However, the long-term health implications of environmental exposure to cadmium in different life stages remain unclear.

**Objectives:**

We investigated the effects of early exposure to cadmium, at an environmentally relevant dosage, on adult metabolism and the mechanism of action.

**Methods:**

We established mouse models with low-dose cadmium (LDC) exposure in early life to examine the long-term metabolic consequences. Intestinal flora measurement by 16S rDNA sequencing, microbial ecological analyses, and fecal microbiota transplant was conducted to explore the potential underlying mechanisms.

**Results:**

Early LDC exposure (100 nM) led to fat accumulation in adult male mice. Hepatic genes profiling revealed that fatty acid and lipid metabolic processes were elevated. Gut microbiota were perturbed by LDC to cause diversity reduction and compositional alteration. Time-series studies indicated that the gut flora at early-life stages, especially at 8 weeks, were vulnerable to LDC and that an alteration during this period could contribute to the adult adiposity, even if the microbiota recovered later. The importance of intestinal bacteria in LDC-induced fat accumulation was further confirmed through microbiota transplantation and removal experiments. Moreover, the metabolic effects of LDC were observed only in male, but not female, mice.

**Conclusions:**

An environmental dose of cadmium at early stages of life causes gut microbiota alterations, accelerates hepatic lipid metabolism, and leads to life-long metabolic consequences in a sex-dependent manner. These findings provide a better understanding of the health risk of cadmium in the environment.

**Citation:**

Ba Q, Li M, Chen P, Huang C, Duan X, Lu L, Li J, Chu R, Xie D, Song H, Wu Y, Ying H, Jia X, Wang H. 2017. Sex-dependent effects of cadmium exposure in early life on gut microbiota and fat accumulation in mice. Environ Health Perspect 125:437–446; http://dx.doi.org/10.1289/EHP360

## Introduction

Cadmium, a ubiquitous environmental compound, is present in soils, sediments, air, and water. Cadmium exposure affects human populations worldwide. In addition to cigarette smoke, contamination of the food chain by natural and anthropogenic sources is the primary route of cadmium exposure. The tolerable weekly intake (TWI) of cadmium is set at 2.5 μg/kg body weight (BW) by the European Food Safety Authority (EFSA) and at 5.8 μg/kg BW by the Joint FAO/WHO Expert Committee on Food Additives (JECFA) (EFSA 2011; FAO/WHO 2010). However, the estimated dietary cadmium intake is 5.81 μg/kg BW per week in Catalonia, Spain; 4.03 μg/kg BW per week in Bangladesh; and 3.67 μg/kg BW per week in China (Al-Rmalli et al. 2012; Domingo et al. 2012; Yuan et al. 2014). These values are above or near the JECFA/EFSA-established TWI value.

After dietary absorption, cadmium remains in the body with a biological half-life of 10–30 years (Järup and Åkesson 2009). Many human diseases, including renal dysfunction, osteoporosis and fractures, cardiovascular diseases, and cancers are associated with cadmium exposure (Joseph 2009; Ke et al. 2015; Solenkova et al. 2014; Trzcinka-Ochocka et al. 2010). As determined in animal studies, high doses of cadmium lead to nephrosclerosis, osteomalacia, testicular interstitial hemorrhage with edema, fetal death, and placental necrosis (Takiguchi and Yoshihara 2006; Thijssen et al. 2007; Umemura 2000).

The adverse effects of relatively low-dose cadmium exposure on metabolic disorders have been investigated recently. In various populations, metabolic syndrome, diabetes, and nonalcoholic fatty liver disease (NAFLD) are associated with the cadmium burden (Hyder et al. 2013; Lee and Kim 2013; Wallin et al. 2016). In mice, 10 mg/L (~ 54.5 μM) of cadmium in the drinking water causes hepatic metabolic changes consistent with NAFLD (Go et al. 2015). However, considering the levels of exposure and the bioaccumulation of cadmium, the health risk at the concentrations of environmental exposure and the underlying mechanisms need further study.

In addition, cadmium can penetrate the placental barrier to result in fetal exposure (Chen et al. 2014). Pregnant women appear to accumulate more cadmium (Nishijo et al. 2004; Vahter et al. 2002), which is largely retained in the placenta. Prenatal cadmium exposure can be toxic in early life, with growth restriction and immune effects in newborns, and poor health outcomes in later life (Gardner et al. 2013; Kim et al. 2013; Kippler et al. 2012b). Prenatal cadmium disturbs transfer of micronutrients to the fetus, interferes with hormone balance, increases oxidative stress, and alters the epigenetic signatures (Kippler et al. 2010, 2012a; Vilahur et al. 2015; Wang et al. 2014). Nevertheless, other toxicological effects of early-life cadmium exposure, especially the long-term effects in later life, should be investigated.

Gut microbiota, which function in production of nutrients, metabolic processing, breakdown of toxins, and protection from infections, are involved in a host’s health at various life stages. Intestinal dysbiosis may contribute to a variety of diseases (Konkel 2013; Ridaura et al. 2013). Since the homeostasis of microbiota develops in early life, it is vulnerable to external stimuli at this stage (Cox et al. 2014; Cunningham et al. 2014; Rodríguez et al. 2015). Early exposure to chemicals, even at the doses generally recognized as safe, can disturb intestinal flora and result in metabolic disorders later in life (Chassaing et al. 2015; Suez et al. 2014). Therefore, gut microbiota could be used to assess the toxicity of environmental pollutants, particularly at low dosages and during early exposure.

To elucidate the long-term adverse outcomes of early-life cadmium exposure at environmental concentrations, we established a mouse model involving early exposure to 100 nM cadmium (LDC), a dose as low as its TWI. The biological effects of LDC on metabolic phenotypes in adults were explored, and a sex dependence was discovered. The underlying mechanism was evaluated from the perspectives of gut microbiota homeostasis. Moreover, through a time-series study, a critical stage of life for the toxicity of cadmium was identified.

## Methods

### Animal Husbandry

C57BL/6J mice were obtained from the Shanghai Laboratory Animal Center (Shanghai, China) and housed under environmental conditions of 23 ± 3°C, 35 ± 5% humidity, and a 12-hr dark/light cycle (lights on from 0600 to 1800 hours) in a specific pathogen-free animal facility. All mice were fed with a standard laboratory rodent diet irradiated with cobalt-60 (SLRC Laboratory Animal Co. Ltd., Shanghai, China) and water *ad libitum*. Control mice received water alone and LDC-exposed mice received drinking water that dissolved 100 nM cadmium chloride (Sigma Aldrich Inc., St. Louis, MO). For fertility, female mice (at 8 weeks of age) exposed by LDC for 1 week were individually caged with age-matched males. After copulation, the male mice were removed and the pregnant and lactating mice were individually caged. Their offspring were separately housed (4–6 mice per cages) after weaning. For antibiotic treatment, weaned mice were given both ciprofloxacin (0.2 g/L) and metronidazole (1 g/L) (Sigma Aldrich) in drinking water. All animals were treated humanely and with regard for alleviation of suffering according to the Biomedical Research Ethics Committee of Shanghai Institutes for Biological Sciences, Chinese Academy of Science (SIBS, CAS).

### Body Composition

The body fat, lean, and total mass of mice were determined by a nuclear magnetic resonance spectroscopy (NMR) with a Minispec Mq7.5 Analyzer (Bruker, Germany).

### Enzyme-Linked Immunosorbent Assay (ELISA)

Plasma levels of total cholesterol (TC), high-density lipoprotein (HDL), very low-density lipoprotein (VLDL) and low-density lipoprotein (LDL) were determined with the corresponding ELISA Kits (BioAssay, Hayward, CA) following the manufacturer’s instructions. Liver and plasma levels of triglycerides (TG) were measured by the Triglyceride Quantification Colorimetric/Fluorometric Kit (BioVision, Milpitas, CA). Plasma leptin was measured with the Mouse Leptin ELISA Kit (Millipore, Darmstadt, Germany). Plasma free fatty acids were determined with the Free Fatty Acid Quantification Kit (Abcam, Cambridge, UK) according to the manufacturer’s instructions.

### Liver Histology

Oil Red O staining was accomplished after liver tissues were fixed in methanol-free 4% paraformaldehyde for 24 hr. A subset of fixed livers was dehydrated in 20% and 30% sucrose equilibration for 12 hr each at 4°C and then cryoembedded in optimal cutting temperature medium (OCT). Cryostat sections (6 μm thick) were prepared and stained with 0.5% Oil Red O (Sigma Aldrich Inc.) for 8 min at room temperature. Staining images were made by use of a light microscope (Olympus, Tokyo, Japan).

### Hepatic Gene Expression Profiling

Total RNA was extracted from 30-week-old mouse livers (*n* = 3 for each group) with Takara RNAiso Plus Kits (Takara, China). RNA quality was assessed by an Agilent Bioanalyzer 2100 (Agilent Technologies, Santa Clara, CA) and further purified by RNeasy Mini Kits (QIAGEN, GmBH, Germany) and RNase-Free DNase Sets (QIAGEN). Microarray analysis with the Affymetrix Genechip Mouse Genome 430 2.0 system (Affymetrix, Santa Clara, CA) was performed according to the standard Affymetrix protocol in Shanghai Biotechnology Corporation. Raw data were normalized by the MAS 5.0 algorithm in GeneSpring software (version 12.6.1; Agilent Technologies). Of 34,000 annotated genes, 104 genes in male mice liver and 68 genes in female mice liver, for which expression levels were at least 2-fold different with *p*-value < 0.05 between control and LDC mice, were selected for analyses. Gene expression heatmaps were constructed using TreeView (version 1.1.1; http://jtreeview.sourceforge.net/). The up-regulated differential genes in male LDC mice were used to perform gene oncology (GO) enrichment (*p* < 0.05, FDR < 0.05) and construct the network of GO biological processes in Cytoscape software (version 3.3.0) with the BiNGO plugin (version 3.0.2).

### Microbial Community Analysis

Total bacteria genomic DNA extraction was performed from fecal pellets and cecal specimens by use of the E.Z.N.A.® Stool DNA Kits (Omega Bio-tek, Norcross, GA). The microbial 16S rDNA was amplified with indexes and adaptors-linked universal primers (341F: ACT​CCT​ACG​GGA​GGC​AGC​AG, 806R: GGA​CTA​CHV​GGG​TWT​CTA​AT) targeting the V3-4 region, purified with QIAquick PCR Purification Kit (QIAGEN), and quantified by Qubit 2.0 Fluorometer (Thermo Fisher Scientific, Waltham, MA) to pool into even concentration. Amplicon libraries were sequenced on Illumina Miseq platform (Illumina, San Diego, CA) for paired-end reads of 300 bp. The paired-end reads were assembled into longer tags and quality filtered to remove tags with length of < 220 nt, average quality score of < 20, and tags containing > 3 ambiguous bases by PANDAseq. After discarding the singletons, the high-quality tags were clustered into operational taxonomic units (OTUs) using Usearch in QIIME software with a similarity threshold of 0.97. And the OTUs were further subjected to the taxonomy-based analysis by RDP algorithm using Greengenes database (http://greengenes.lbl.gov). Alpha diversity (Shannon) and beta diversity [weighted UniFrac, principal coordinate analysis (PCoA)] were analyzed using QIIME. Linear discriminant analysis (LDA) effect size (LEfSe) analyses were performed with the LEfSe tool (http://huttenhower.sph.harvard.edu/galaxy).

### Fecal Microbiota Transplant

For donors, male mice (control, *n* = 8; LDC, *n* = 10) received water alone or 100 nM LDC continuously beginning at fertilization and throughout the transplantation. Starting from 3 weeks of age, the fecal microbiota of donor mice was collected and transferred to the age-matched recipient mice. Briefly, the stools from control or LDC donors were pooled in sterile saline (100 mg/mL), resuspended by vigorous mixture for 30 sec, and centrifugalized at 800 *g* for 3 min. The supernatant was collected and delivered to the recipient mice via oral gavage (100 μL each recipient) within 10 min to prevent changes in bacterial composition. The recipient mice (*n* = 8 each group), which were fed with normal diet and drinking water, were subjected to the microbiota transplant twice a week from 3 weeks of age.

### Statistical Analyses

The body composition and metabolic results were presented as means ± SD, and the significant differences were examined using ANOVA or Student’s *t*-test. Differences in gut bacterial abundance were analyzed by the Mann–Whitney *U* test (version 6.0.1; GraphPad Prism). For the LEfSe analysis, we used the Kruskal–Wallis rank sum test to detect significantly different abundances and performed LDA scores to estimate the effect size (threshold: ≥ 2). A *p-*value < 0.05 was considered statistically significant.

## Results

### Effect of Early-Life LDC Exposure on Adult Body Composition

**Figure 1:**
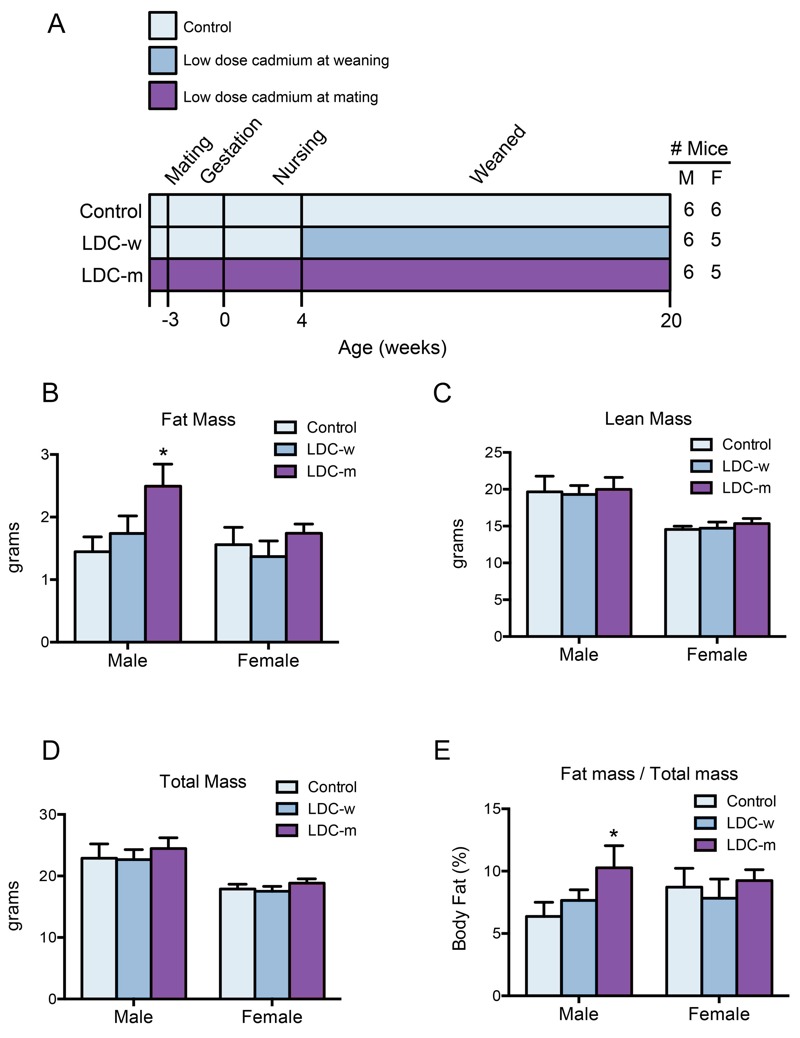
Effect of early-LDC exposure on body composition. (*A*) Study design: C57BL/6J mice received LDC (100 nM in drinking water) continuously for life beginning either at weaning (day 28, LDC-w, *n *= 6 males and *n *= 5 females), or from fertilization (LDC-m, *n *= 6 males and *n *= 5 females). Control mice received water alone (*n *= 6 males and *n *= 6 females). (*B*–*D*) Body composition was measured by NMR at week 20; *p < 0.05 compared with the control. (*E*) Body fat percentage in male and female mice was measured; *p < 0.05 compared with the control. Note: F, female; M, male.

To investigate the toxicological effects of LDC in early-life stages, a series of concentrations of cadmium in drinking water (0.02 to 500 nM) were provided to maternal mice throughout their pregnancy. Thus, the offspring were exposed to cadmium from the initial stage of life. Although body lengths of newborn mice were similar, cadmium-exposed mice in the 100 and 500 nM groups had lower body weights compared with the control group (see Figure S1A,B). Next, 100 nM cadmium was employed to establish the LDC mouse model for early-life exposure. C57BL/6J mice received LDC continuously for life either beginning at weaning (LDC-w), or 1 week before the parental mating (LDC-m); control mice received drinking water only (Figure 1A). At adulthood (20 weeks), values for the fat mass and percent body fat were significantly increased in LDC-m male mice (Figure 1B and E); the lean and total masses showed little difference (Figure 1C,D). The body compositions in LDC-w male mice showed similar trends but no significant difference (Figure 1B–E). These results indicated that LDC led to fat accumulation in male mice and that the exposure before weaning is more effective. Of note, the promoting effect of LDC on body fat was observed only in male mice. Neither female LDC-w nor female LDC-m mice had elevated fat mass compared with controls (Figure 1B–E).

### Effect of Early-Life LDC Exposure on Adiposity Metabolism

**Figure 2:**
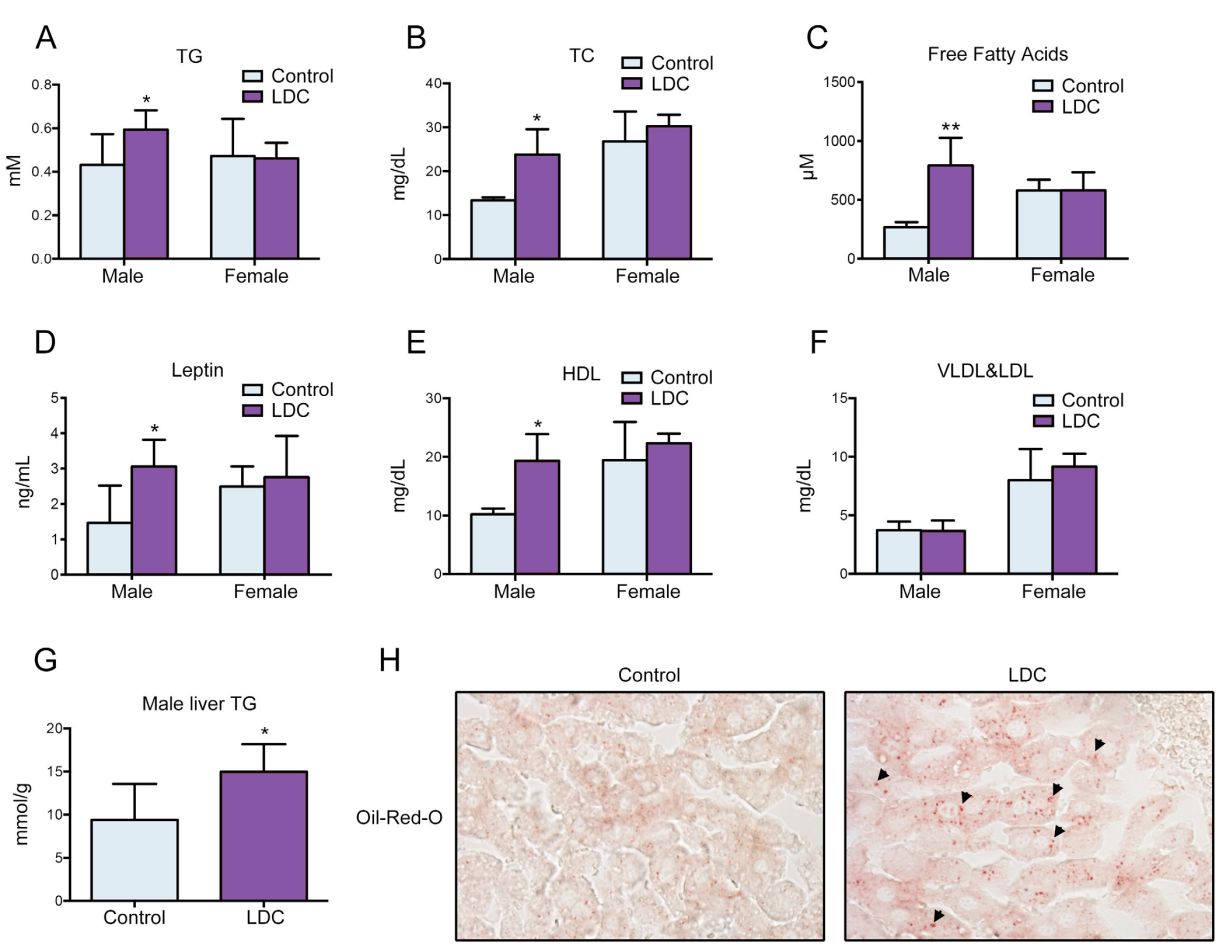
Early-life LDC exposure disturbed lipid homeostasis. (*A*–*F*) The plasma levels of TG (*A*), TC (*B*), free fatty acids (*C*), leptin (*D*), HDL (*E*) and VLDL&LDL (*F*) in male and female mice were measured at week 30; *p < 0.05, **p < 0.01 compared with the control group. (*G*) The contents of liver TG in male mice was measured at week 30; *p < 0.05 compared with the control group. (*H*) Representative images of Oil Red O staining of liver sections from control and LDC male mice. The arrows indicate the lipid deposits in liver cells. Magnification, ×40.

Based on the above results, we used LDC-m mice as the LDC model in the following studies. The effect of LDC on fat accumulation was validated by determining the lipid levels in plasma. LDC early exposure significantly elevated plasma TG and TC levels in male but not in female mice (Figure 2A,B). The plasma levels of free fatty acids in LDC male mice were also higher than those in control group (Figure 2C). Thus, early exposure to LDC facilitates a general effect on levels of blood lipids. Further, the levels of leptin, a hormone secreted by adipocytes, and HDL, which transports fat molecules to the liver, were increased by LDC (Figure 2D,E); whereas, the VLDL and LDL levels were unaffected (Figure 2F), suggesting that the metabolism related to body adiposity is dysregulated by LDC exposure. In regard to the accumulation of fat in liver tissue, LDC resulted in an increase of liver TG in male mice (Figure 2G) and lipid deposits in liver cells (Figure 2H). No other abnormalities relating to energy metabolism, such as fasting blood glucose, food intake, or body weight, were observed.

### LDC-Induced Differentially Expressed Genes in Liver

**Figure 3:**
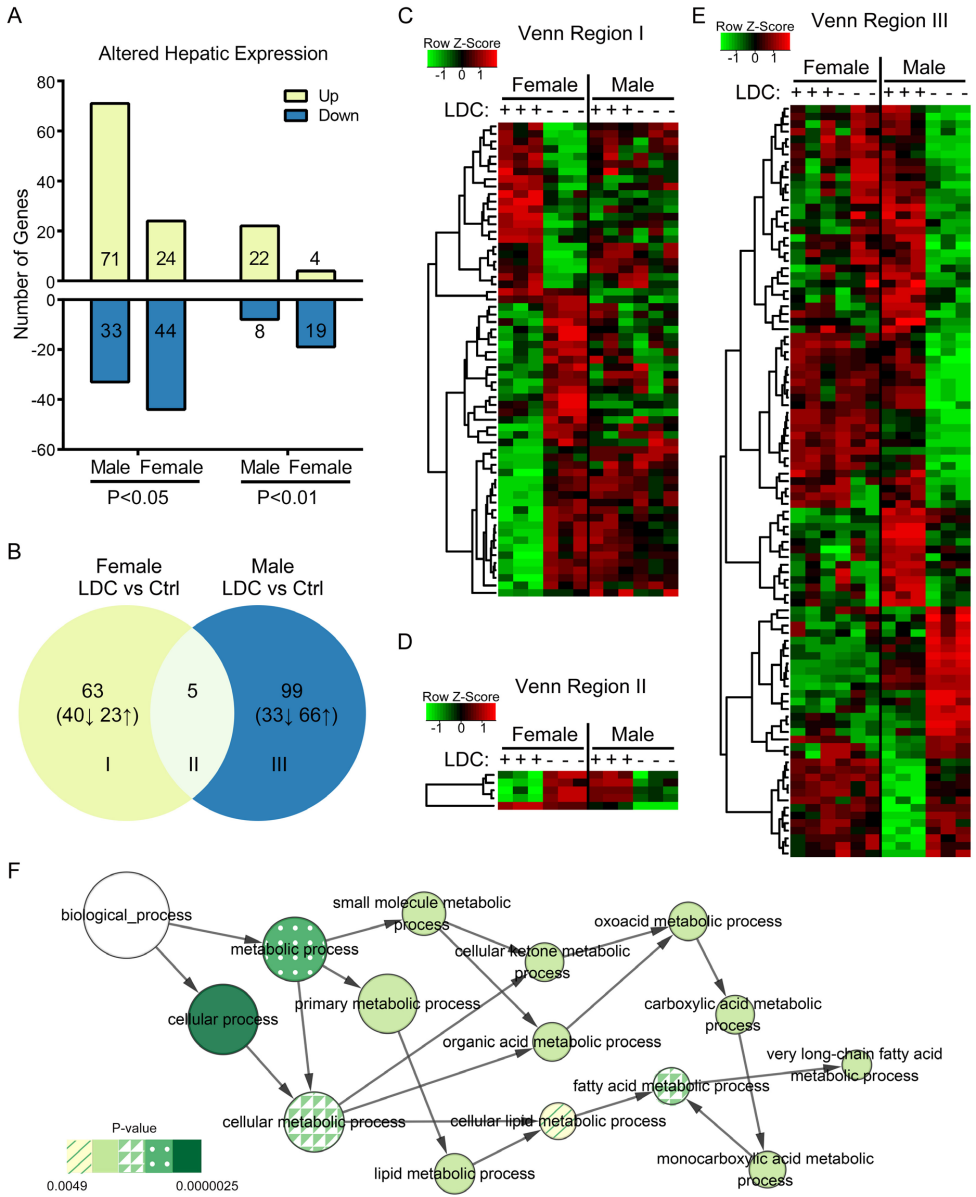
Effect of early-life LDC exposure on hepatic gene expressions. (*A*) Number of LDC-induced differentially expressed hepatic genes (*p* < 0.05 or 0.01, fold change ≥ 2) in male and female mice. (*B*) The differential expression of hepatic genes was comparatively analyzed. A Venn diagram represents the numbers of overlapping genes between two separate pairwise comparisons: male mice [LDC vs. Control (Ctrl)] and female mice (LDC vs. Control). (*C*–*E*) The expression values of genes in Venn regions I–III were shown in heatmaps. (*F*) The lipid metabolism-related biological processes enriched by up-regulated differential genes in LDC male mice were represented in the GO analysis network. Node size shows the number of genes annotated in each GO term and the node color represents the significance of the enrichment.

To further investigate the LCD-induced adiposity and its sex dependence, hepatic gene expression profiling analysis was performed by a microarray (GEO accession number: GSE83326). LDC exposure had a stronger effect on hepatic gene expression in adult male mice than in adult female mice at the age of 30 weeks (Figure 3A). Moreover, LDC induced more up-regulations of genes in male mice (71/104) but more down-regulations in female mice (44/68). Through comparative analysis of significantly differentially expressed genes, male- or female-specific LDC-altered genes were identified (Figure 3B–E). Only 5 genes were regulated by LDC both in male and female mice; however, 4 of these showed a contrary trend (Figure 3D), indicating that the mice of different sexes responded to early-LDC exposure in distinct ways.

The differentially expressed gene sets were then analyzed for enrichment in GO biological process annotations. In male but not female mice, LDC enhanced the expression of genes involved in functions linked to fatty acid and lipid metabolism (Figure 3F; see also Table S1), which is consistent with the phenotypic changes and with the differential effect of LDC on males and females.

### Dynamics of LDC-Induced Adiposity and Microbiota Homeostasis

**Figure 4:**
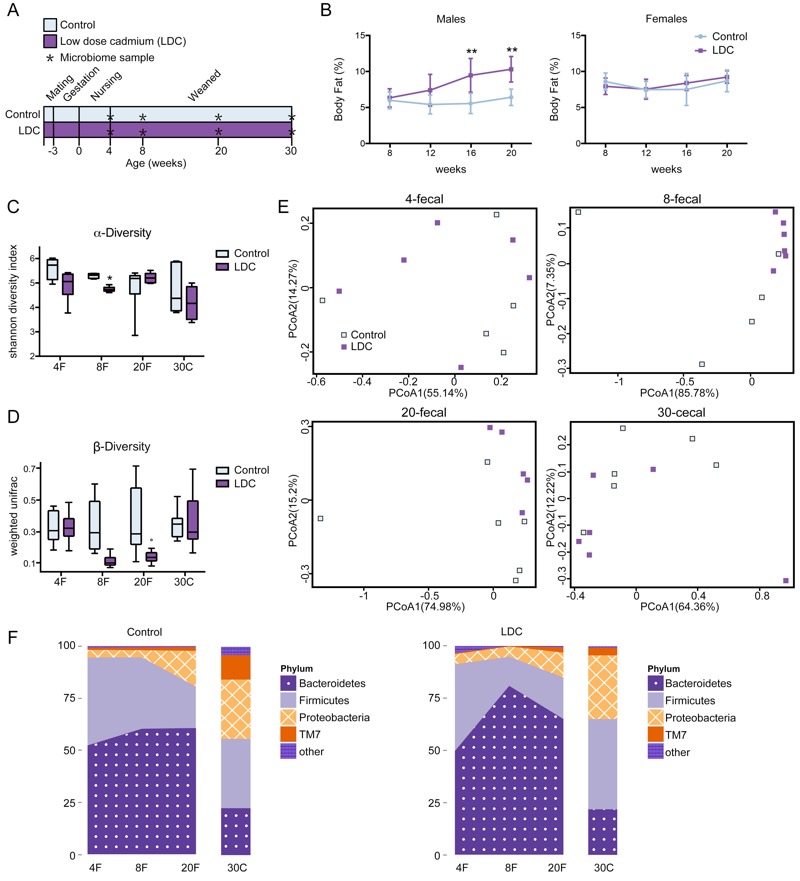
Dynamics of adiposity and microbiota changes after LDC exposure. (*A*) Study design: C57BL/6J mice received LDC (*n *= 6 males and *n *= 5 females) or only water (*n *= 6 males and *n *= 6 females) from fertilization to 30 weeks of age. (*B*) Body fat percentage in male and female mice was measured over time by magnetic resonance spectroscopy (NMR); **p < 0.01 compared with the control group. (*C*-*F*) Fecal specimens at weeks 4, 8, and 20, and the terminal (week 30) cecal specimens from male mice were collected and the intestinal floras were examined by 16S rDNA sequencing. (*C*) Alpha diversity of control and LDC community was analyzed by Shannon diversity index; *p < 0.05 compared with the control group. (*D*) Intragroup β-diversity of control and LDC community was measured by weighted UniFrac distance. (*E*) The plots generated by the weighted UniFrac-based PCoA at each time point. (*F*) LDC affected the relative abundance of predominant bacteria at the phylum level in control and LDC male mice. Note: PCoA1 and PCoA2 are the two dimensions with the most significant differences in the analysis.

For detailed pattern of toxicity, time-series studies were conducted to obtain a dynamic view of LDC-mediated phenotype development (Figure 4A). Male LDC mice showed significant fat accumulation beginning at 16 weeks of life, but there was already a trend toward accumulation at 12 weeks (Figure 4B). No significant change of body fat occurred in female LDC mice (Figure 4B).

The effects of LDC on gut microbiota were determined by 16S rDNA sequence-based analysis of fecal pellets (weeks 4, 8, and 20) and cecal specimens (week 30) in male mice. The alpha diversity of microbial community indicated by the Shannon index was reduced significantly by LDC at 8 weeks of life, and it recovered afterward (Figure 4C). Moreover, compared with control group, microbiota community in mice exposed to LDC exhibited lower intragroup beta diversity from the 8th week until 20th week, which was measured by weighted UniFrac distances (Figure 4D). The weighted UniFrac-based PCoA revealed distinct clusters of microbiota composition in LDC mice at 8 and 20 weeks (Figure 4E). Notably, taxonomic profiling demonstrated that the microbiota pattern of succession in early life was altered by LDC, particularly at 8 weeks (Figure 4F). These results suggest that the first 8 weeks is likely to be a critical window for gut microbiota development and that a disturbance in this period could contribute to the adiposity that occurs later.

### Alteration of Gut Microbiota Composition at 8 Weeks

**Figure 5:**
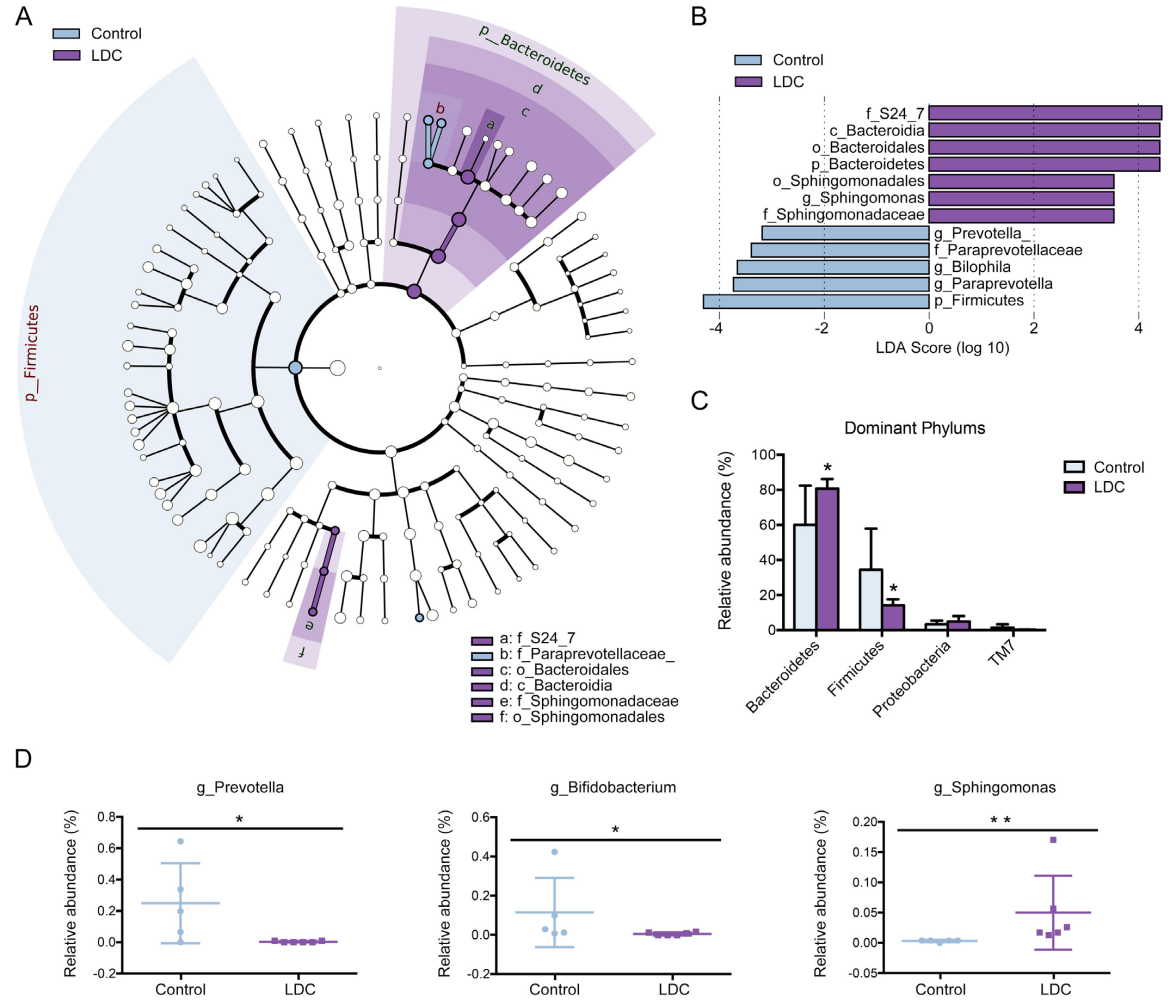
Characteristics of microbial community composition in 8-week male mice. (*A*) The enriched taxa in control and LDC 8-week fecal microbiota were represented in Cladogram. The central point represents the root of the tree (Bacteria), and each ring represents the next lower taxonomic level (phylum to genus: p, phylum; c, class; o, order; f, family; g, genus). The diameter of each circle represents the relative abundance of the taxon. (*B*) The most differentially abundant taxa between control and LDC groups were identified through the LDA score which was generated from LEfSe analysis. (*C*) Relative abundance of dominant phyla was compared between control and LDC groups; *p < 0.05 significantly different by Mann–Whitney *U* test. (*D*) Relative abundance at the bacterial genus level between control and LDC groups was compared; *p < 0.05, **p < 0.01 significantly different by Mann–Whitney *U* test.

We further assessed the LDC-induced alterations in microbiota composition at 8 weeks. To explore the specific bacterial taxa associated with early LDC exposure, a LEfSe comparison of the gut microbiota between control and LDC communities was performed. The structure and predominant bacteria of microbiota in control and LDC male mice (week 8) was represented in a cladogram (Figure 5A). The greatest difference in taxa from phylum to genus level was identified via LDA score (Figure 5B). Most of the specific taxa fell in two most dominant phyla, Bacteroidetes and Firmicutes. At the phylum level, the abundance of Bacteroidetes was increased and Firmicutes was diminished by LDC (Figure 5C). At the genus level, LDC exposure in male mice significantly decreased the levels of *Bifidobacterium* and *Prevotella*, which utilize carbohydrates, especially plant-derived oligosaccharides and polysaccharides (Figure 5D). A bacterium that can absorb and accumulate cadmium, *Sphingomonas*, was more abundant in LDC mice (Figure 5D). Notably, the LDC-induced alteration of gut microbiota composition at 8 weeks was unique to male mice; no corresponding changes were evident in 8-week female LDC mice (see Figure S2A–C). These results further explain the sex dependence of LDC-induced fat accumulation.

### Essentiality of Gut Microbiota in Fat Accumulation

**Figure 6:**
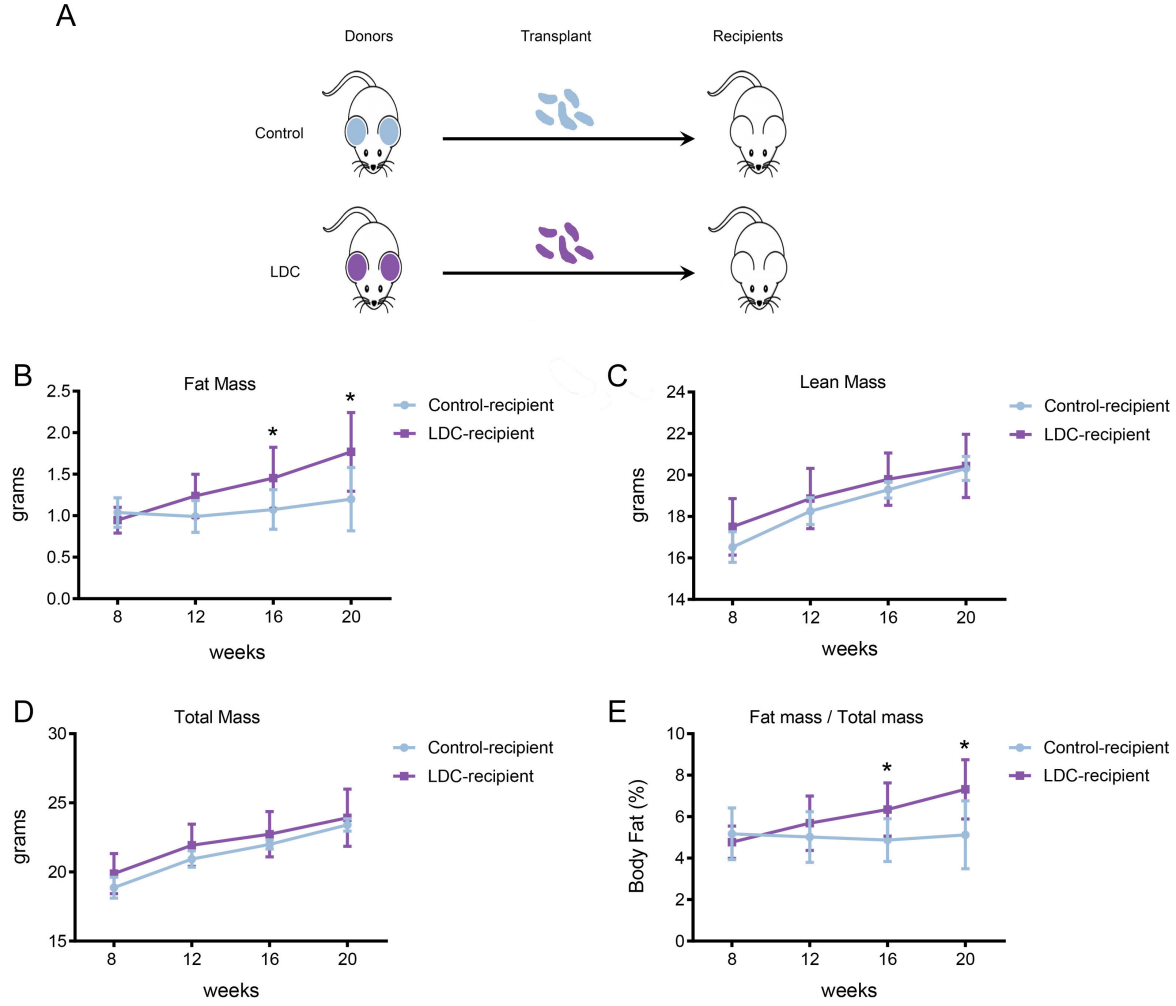
Effect of microbiota transplant on body compositions. (*A*) Study design: 3-week-old control or LDC male mice (control, *n *= 8; LDC, *n *= 10) were used as donors. The fecal contents were collected, pooled, and transferred to age-matched male recipient mice (*n *= 8 for each group) by oral gavage twice a week. No recipient mice received LDC during the experiment. (*B*–*D*) Body composition in the control- and LDC-microbiota recipient mice was measured by NMR at 8, 12, 16, and 20 weeks of age. *p < 0.05 compared with the control-recipient group. (*E*) Body fat percentage in control- and LDC-microbiota recipient mice was measured over time. *p < 0.05 compared with the control-recipient group.

To determine the causality of altered microbiota, we transferred fecal microbiota from male control or LDC mice to the age-matched male recipient mice (not exposed to cadmium) (Figure 6A). The mass (g) and proportion (%) of body fat in LDC-microbiota recipient mice were increased compared with that in control-recipients; no significant changes were detected in lean and total mass (Figure 6B–E) These results indicate that the host metabolic changes are driven by the LDC-altered microbiota.

Moreover, we treated male control and LDC mice with a Gram-negative targeting broad-spectrum antibiotics regimen (ciprofloxacin and metronidazole) after weaning (see Figure S3A). Notably, after antibiotic treatment, the elevation of body fat in LDC mice compared with controls was abolished. Both the fat mass and the percentage of fat were decreased in LDC mice, even though the lean and total mass were reduced (see Figure S3B–E). Taken together, these findings suggest that LDC-induced fat accumulation is mediated by the alterations of commensal microbiota.

## Discussion

In this study, a mouse model developed for early-life exposure to LDC showed that LDC exposure from the very beginning of life led to increased adiposity in male but not female mice. In liver tissues, LDC also elevated the expressions of genes related to fatty acid and lipid metabolism. Early life is critical for gut microbiota development. LDC-induced alterations of gut microbiota in early stages of life could lead to accumulation of fat in adults, even if the bacterial dysbiosis was recovered later.

Previous toxicity studies of cadmium have focused on kidney and bone damage, and epidemiologic evidences have linked low-level cadmium exposure with adverse effects including diabetes, hypertension, peripheral artery disease, and cancers (Adams et al. 2014; Edwards and Ackerman 2016; Eum et al. 2008; Julin et al. 2012; Navas-Acien et al. 2005; Romano et al. 2015). However, in functional studies based on animal models, the dosage of cadmium was much higher than the mean intake of populations (Du et al. 2015; L Liu et al. 2015; Y Liu et al. 2014). In this work, we used 100 nM cadmium in drinking water, which is equivalent ~ 2.5 μg/kg BW per week and closely corresponds to the TWI and the mean intake by humans. Such low dose of cadmium was still found to modulate body composition, hepatic gene expression, and intestinal microbiota, implying a possible universal impact.

The timing of LDC exposure relates to its toxicological effects. It is known that cadmium is embryotoxic and teratogenic (del C Díaz et al. 2014), which is confirmed by our results that maternal cadmium exposure (100 nM in drinking water) during pregnancy affected embryo development by reducing the birth size. During lactation, cadmium obtained through breast milk can disturb development of infants (Cherkani-Hassani et al. 2016; Halder et al. 2016). However, the correlation between early-life exposure and the adult phenotypes is limited. We for the first time observed that early life is the critical window for LDC exposure that results in metabolic consequences. LDC-m mice showed a significant increase in adiposity at 12–16 weeks of age, whereas post-weaning exposure caused no appreciable metabolic changes even at 20 weeks.

Disruption of microbiota in early life affects long-term metabolic programming (Cox et al. 2014; Rodríguez et al. 2015). The present study highlights the possibility that early onset LDC-induced gut microbiota alterations or dysbiosis contributes to metabolic abnormalities in adults. The diversity reduction and compositional changes of microbial communities occurred at 8 weeks of age, prior to fat accumulation (week 12–16), suggesting that alteration of microbiota is an upstream event that drives the later metabolic effects. The causality of intestinal bacteria was further validated by fecal microbiota transplant and simulated germ-free mice experiments. Besides, the key microbiota characteristics in LDC mice were identified. First, LDC exposure was associated with reduced microbiota abundance and taxonomic diversity in male mice, which is considered to be involved in obesity-related metabolic pathways. Second, LDC-induced disruption of microbiota homeostasis in male mice occurred most prominently at 8 weeks of life. The microbiota generally recovered later but the fat accumulation persisted. Third, compositional differences of microbiota also contributed to LDC-induced adiposity. *Prevotella* and a probiotic bacterium, *Bifidobacteria*, were diminished; these changes are similar to those in obese individuals (Cani et al. 2008; Cox et al. 2014).

The findings indicate that LDC reduces the diversity of the gut microbiota, particularly decreases early-life protective bacterial populations, and subsequently resets the metabolic programming throughout the life. Perturbations of intestinal microbiota may be a mechanism by which cadmium exposure leads to human metabolic diseases. Future studies are needed to confirm the role of cadmium-induced changes in bacteria and its metabolites in humans, and to identify particular species and strains that are targeted by cadmium exposure. Solving these issues could lead to development of probiotics strategies for eliminating the health hazards of cadmium pollution.

LDC induced phenotypic development with notable sex differences. Early exposure of LDC caused adiposity only in male mice, which is consistent with sex-dependent correlation found in epidemiological studies (Hyder et al. 2013; Lee and Kim 2013). Although cadmium disrupts the endocrine system by targeting the estrogen pathway (Fechner et al. 2011), male animals have been generally chosen for metabolic studies related to cadmium exposure (Go et al. 2014, 2015). Thus, the sex dependence of cadmium toxicity is less investigated. Multiple factors may contribute to this sex difference. It is reported that cadmium exposure during pregnancy could alter fetal DNA methylation in a sex-specific manner (Kippler et al. 2013). In our study, the changes of hepatic gene expression profiles totally differ between cadmium-exposed male and female mice. Only one common gene variation was found in male and female LDC mice. GO-based functional analysis showed that the enriched processes were concentrated mainly in fatty acid metabolism and organic development in male mice; in contrast, pathways related to external stimuli reaction, immunity, and ion were influenced in female mice. Further, consistent with the metabolic variation, gut microbiota in male and female hosts responded differently to LDC exposure. Obesity-related bacteria in males were more sensitive to LDC. In sum, these findings demonstrated the sex differences in cadmium toxicity and provided possible explanations for these effects.

## Conclusions

In summary, an environmental dose of cadmium during early development causes, in a sex-dependent manner, changes of gut microbiota, a disorder in hepatic lipid metabolism, and accumulation of fat in adults. The alteration of gut microbiota is a novel mechanism by which cadmium exposure can lead to metabolic consequences. Moreover, modulation of gut microbiota could serve as a potential intervention to protect against the chronic effects of cadmium toxicity.

## Supplemental Material

(323 KB) PDF
