# Supplementary material for: Sex-Dependent Effects of Cadmium Exposure in Early Life on Gut Microbiota and Fat Accumulation in Mice
Source: Environ Health Perspect. 2016 Sep 16;125(3):437–46. doi: 10.1289/EHP360 (PMC5332190; doi:10.1289/EHP360)
Supplement: (323 KB) PDF [file EHP360.s001.acco.pdf]

Environ Health Perspect
DOI: 10.1289/EHP360

Note to readers with disabilities: *EHP* strives to ensure that all journal content is accessible to all readers. However, some figures and Supplemental Material published in *EHP* articles may not conform to 508 standards due to the complexity of the information being presented. If you need assistance accessing journal content, please contact ehponline@niehs.nih.gov. Our staff will work with you to assess and meet your accessibility needs within 3 working days.

Supplemental Material

Sex-Dependent Effects of Cadmium Exposure in Early Life on Gut Microbiota and Fat Accumulation in Mice

Qian Ba, Mian Li, Peizhan Chen, Chao Huang, Xiaohua Duan, Lijun Lu, Jingquan Li, Ruiai Chu, Dong Xie, Haiyun Song, Yongning Wu, Hao Ying, Xudong Jia, and Hui Wang

Table of Contents

Figure S1. Effect of Prenatal Cadmium Exposure on Size at Birth. Female C57BL/6J mice received LDC (0.2, 2, 10, 20, 100, or 500 nM in drinking water) a week before mating and throughout the pregnancy. The body weights (A) and body lengths (B) of the pups were measured at birth. *, $p < 0.05$; **, $p < 0.01$ compared with the control.

Figure S2. Intestinal Bacteria changes at the genus level in 8-week-old female mice. The fecal microbiota in control and LDC female mice at 8 weeks of age were analyzed and the relative abundance of *Prevotella* (A), *Bifidobacterium* (B) and *Sphingomonas* (C) was shown.

Figure S3. Effects of LDC on body compositions in male mice with microbiota removal. (A) Study design: After weaning (3 weeks old), control or LDC male mice (control, n=6; LDC, n=4) were treated with ciprofloxacin (0.2 g/L) and metronidazole (1 g/L) in their drinking water. (B-D) Body composition in the control and LDC mice with antibiotics treatment was measured by NMR at 16 weeks of age. **, $p < 0.01$ compared with the control group.

Table S1. The GO terms of biological process enriched by up-regulated differential genes in LDC male mice.

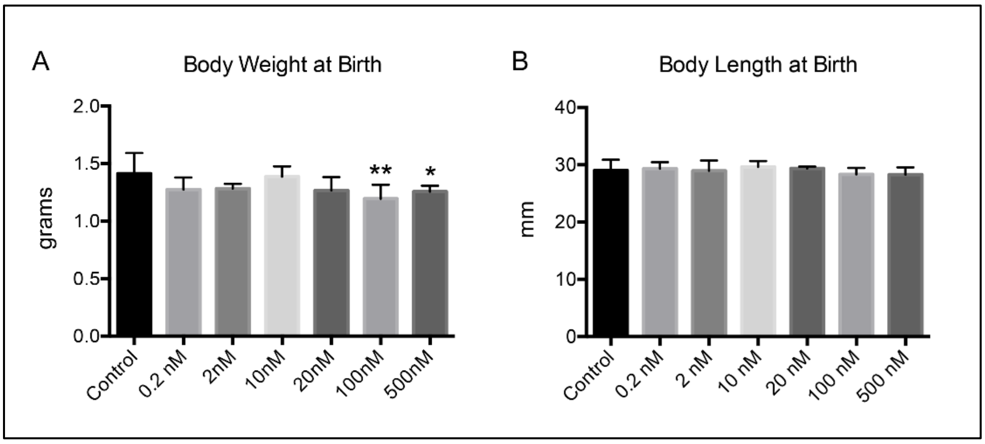

Figure S1. Effect of Prenatal Cadmium Exposure on Size at Birth. Female C57BL/6J mice received LDC (0.2, 2, 10, 20, 100, or 500 nM in drinking water) a week before mating and throughout the pregnancy. The body weights (A) and body lengths (B) of the pups were measured at birth. *, $p < 0.05$; **, $p < 0.01$ compared with the control.

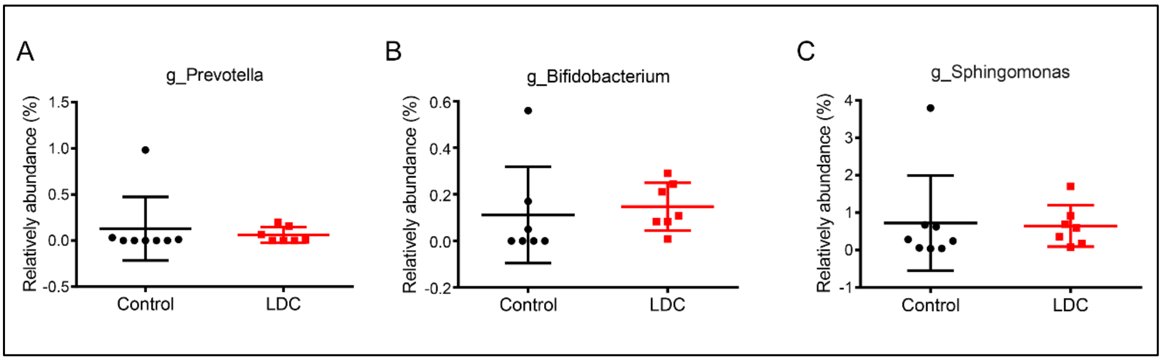

Figure S2. Intestinal Bacteria changes at the genus level in 8-week-old female mice. The fecal microbiota in control and LDC female mice at 8 weeks of age were analyzed and the relative abundance of *Prevotella* (A), *Bifidobacterium* (B) and *Sphingomonas* (C) was shown.

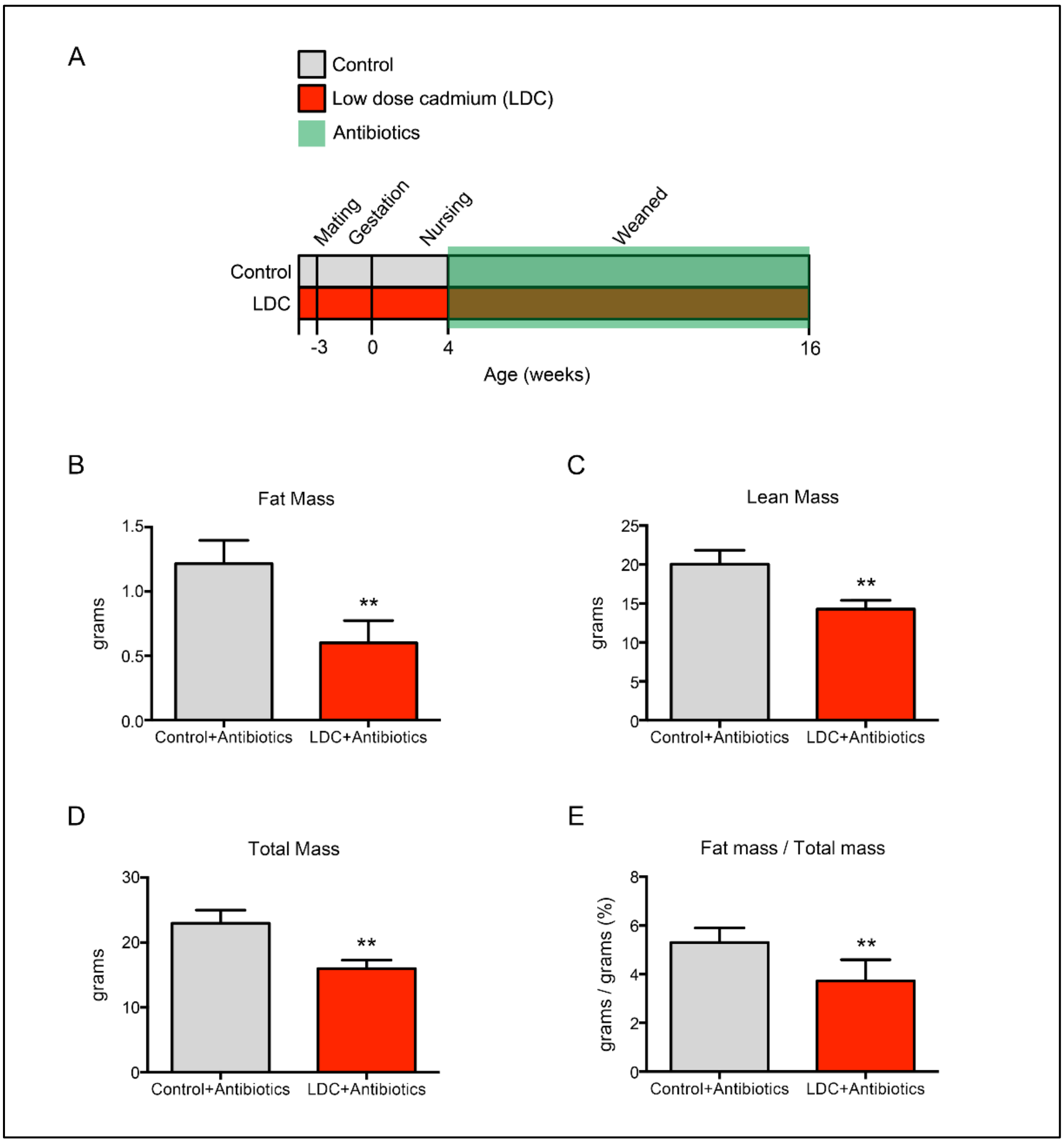

Figure S3. Effects of LDC on body compositions in male mice with microbiota removal. (A) Study design: After weaning (3 weeks old), control or LDC male mice (control, n=6; LDC, n=4) were treated with ciprofloxacin (0.2 g/L) and metronidazole (1 g/L) in their drinking water. (B-D) Body composition in the control and LDC mice with antibiotics treatment was measured by NMR at 16 weeks of age. **, $p < 0.01$ compared with the control group. (E) Body fat percentage in control and LDC mice with antibiotics treatment was measured. **, $p < 0.01$ compared with the control group.

Table S1. The GO terms of biological process enriched by up-regulated differential genes in LDC male mice.

GO-ID	*P*-value	FDR	Description
9987	2.49E-06	1.70E-03	cellular process
8152	1.85E-05	6.34E-03	metabolic process
6631	7.03E-05	1.50E-02	fatty acid metabolic process
61180	8.73E-05	1.50E-02	mammary gland epithelium development
44237	1.63E-04	2.23E-02	cellular metabolic process
60736	3.46E-04	3.96E-02	prostate gland growth
32787	4.34E-04	4.15E-02	monocarboxylic acid metabolic process
60749	6.75E-04	4.15E-02	mammary gland alveolus development
6629	7.45E-04	4.15E-02	lipid metabolic process
44238	8.11E-04	4.15E-02	primary metabolic process
19752	8.38E-04	4.15E-02	carboxylic acid metabolic process
43436	8.38E-04	4.15E-02	oxoacid metabolic process
30879	8.43E-04	4.15E-02	mammary gland development
6082	8.47E-04	4.15E-02	organic acid metabolic process
42180	9.62E-04	4.40E-02	cellular ketone metabolic process
38	1.11E-03	4.60E-02	very long-chain fatty acid metabolic process
6637	1.21E-03	4.60E-02	acyl-CoA metabolic process
35383	1.21E-03	4.60E-02	thioester metabolic process
60560	1.31E-03	4.73E-02	developmental growth involved in morphogenesis
